# PCG Classification Using Multidomain Features and SVM Classifier

**DOI:** 10.1155/2018/4205027

**Published:** 2018-07-09

**Authors:** Hong Tang, Ziyin Dai, Yuanlin Jiang, Ting Li, Chengyu Liu

**Affiliations:** ^1^Department of Biomedical Engineering, Dalian University of Technology, Dalian, China; ^2^College of Information and Communication Engineering, Dalian Minzu University, Dalian, China; ^3^School of Instrument Science and Engineering, Southeast University, Nanjing, China

## Abstract

This paper proposes a method using multidomain features and support vector machine (SVM) for classifying normal and abnormal heart sound recordings. The database was provided by the PhysioNet/CinC Challenge 2016. A total of 515 features are extracted from nine feature domains, i.e., time interval, frequency spectrum of states, state amplitude, energy, frequency spectrum of records, cepstrum, cyclostationarity, high-order statistics, and entropy. Correlation analysis is conducted to quantify the feature discrimination abilities, and the results show that “frequency spectrum of state”, “energy”, and “entropy” are top domains to contribute effective features. A SVM with radial basis kernel function was trained for signal quality estimation and classification. The SVM classifier is independently trained and tested by many groups of top features. It shows the average of sensitivity, specificity, and overall score are high up to 0.88, 0.87, and 0.88, respectively, when top 400 features are used. This score is competitive to the best previous scores. The classifier has very good performance with even small number of top features for training and it has stable output regardless of randomly selected features for training. These simulations demonstrate that the proposed features and SVM classifier are jointly powerful for classifying heart sound recordings.

## 1. Introduction

Heart sounds are a series of mechanical vibrations produced by the interplay between blood flow and heart chambers, valves, great vessels, etc. [1–3]. Heart sounds provide important initial clues in heart disease evaluation for further diagnostic examination [4]. Listening to heart sounds plays an important role in early detection for cardiovascular diseases. It is practically attractive to develop computer-based heart sound analysis. Automatic classification of pathology in heart sounds is one of the hot problems in the past 50 years. But accurate classification is still an open challenge question. To the authors' knowledge, Gerbarg et al. were the first to publish automatic classification of pathology in heart sounds [5].

Automatic classification of PCG recording in clinical application typically consists of four steps: preprocessing, segmentation, feature extraction, and classification. Over the past decades, features and methods for the classification have been widely studied. In summary, features may be wavelet features, time-domain features, frequency domain features, complexity-based features, and joint time-frequency domain features. Methods available for classification may be artificial neural network [6–10], support vector machine [11, 12], and clustering [13–16]. Unfortunately, comparisons between previous methods have been hindered by the lack of standardized database of heart sound recordings collected from a variety of healthy and pathological conditions. The organizers of the PhysioNet/CinC Challenge 2016 set up a large collection of recordings from various research groups in the world. In the conference, many methods were proposed for this discrimination purpose, like deep learning methods [17–19], tensor based methods [20], support vector machine based methods [21, 22], and others [23–27]. Generally, time and/or frequency domain features were used in these papers. The reported top overall scores were 89.2% by [27], 86.2% by [28], 85.9% by [29], and 85.2% by [30]. In this paper, the authors extend their previous study [31] and extracted a total of 515 features for normal/abnormal PCG classification. The difference of the proposed method to the existing methods is that these features are from multidomains, such as time interval, state amplitude, energy, high-order statistics, cepstrum, frequency spectrum, cyclostationarity, and entropy. To the authors' knowledge, the proposed method in this paper perhaps uses the most number of features. Correlation analysis shows the contribution of each feature. A SVM classifier is used to discriminate abnormal/uncertain/normal types. Cross validation shows that the proposed features have excellent generation ability. The mean overall score based on 20% data training is up to 0.84. It rises to 0.87 based on 50% data training and rises to 0.88 based on 90% data training. The results demonstrate that the method is competitive comparison to previous approaches.

## 2. Methods

### 2.1. Database

The database used in this paper is provided by the international competition PhysioNet/CinC Challenge 2016, which can be freely downloaded from the website [32]. The database includes both PCG recordings of healthy subjects and pathological patients collected in either clinical or nonclinical environments. There are a total of 3,153 heart sound recordings, given as “*∗*.wav” format, from 764 subjects/patients, lasting from 5 s to 120 s. The recordings were divided into two classes: normal and abnormal records with a confirmed cardiac diagnosis. Label “1” was used to present abnormal (665 recordings) and “-1” to present normal cases (2488 recordings). A skilled cardiologist was also invited to evaluate the signal quality for each recording. As he believed that a recording had good signal quality, it was labeled “1”. Otherwise, it was labeled “0”. There are 279 recordings which were labeled as bad signal quality and the rest of 2874 were labeled as good quality. Details about the database can be found in [33].

### 2.2. Flow Diagram of the Proposed Method

**Figure 1:**
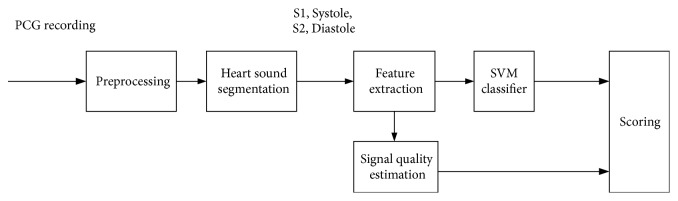
Flow diagram of the proposed classification.

The flow diagram of the proposed method to classify PCG recordings is shown in Figure 1. Each step will be described in the following subsections.

### 2.3. Preprocessing

Each PCG recording is high-pass filtered with a cut-off frequency of 10 Hz to remove baseline drift. The spike removal algorithm is applied to the filtered recording [34]. Then, the recording is normalized to zero mean and unit standard deviation.

### 2.4. Heart Sound Segmentation by Springer's Algorithm

**Figure 2:**
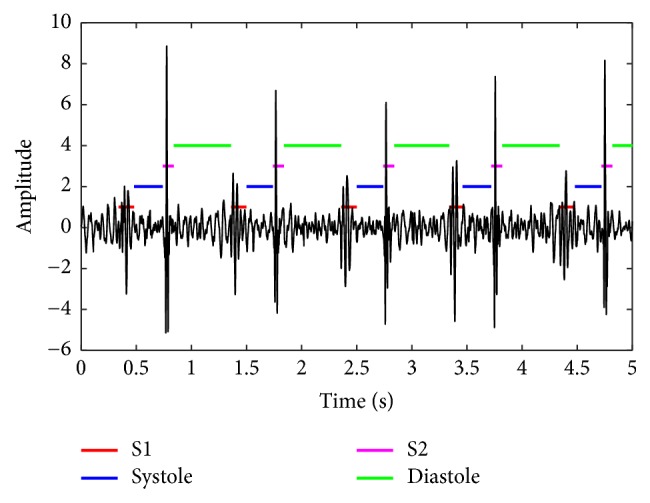
Illustration of the HSMM segmentation.

Springer's hidden semi-Markov model (HSMM) segmentation method [35] is used to segment a PCG recording into four states, i.e., S1, systole, S2, and diastole.  Figure 2 shows an example of this segmentation. Hence, the following signals can be defined and further used for feature extraction. *x*(*n*) is a digital PCG recording where *n* is the discrete time index. *s*_1*i*_(*n*) and *s*_2*i*_(*n*) are S1 and S2 signals occurring in the* i*th cardiac cycle, respectively. *sys*_*i*_(*n*) and *dia*_*i*_(*n*) are the signals of systolic interval and diastole interval in the* i*th cardiac cycle, respectively. *c*_*i*_(*n*) is the signal of the* i*th cardiac cycle. Hence, *c*_*i*_(*n*) consists of the digital sequence of [*s*_1*i*_(*n*)*sys*_*i*_(*n*)*s*_2*i*_(*n*)*dia*_*i*_(*n*)].

### 2.5. Features Extracted in Multidomains

#### 2.5.1. Time-Domain Features (20 Features)

After the segmentation operation, a PCG recording is divided into many states in the order of S1, systole, S2, and diastole. The time interval of each state can be measured by the time difference between the beginning and the end. The cardiac cycle period can be measured by the time difference between the beginnings of two adjacent S1s. Since the intervals have physiological meanings in view point of heart physiology, Liu et al. [33] proposed 16 features from the intervals as shown in Table 1. Another 4 features from time-domain intervals are added in this paper.

#### 2.5.2. Frequency Domain Features for States (77×4=308 Features)

Frequency spectrum is estimated for the S1 state of each cardiac cycle using a Gaussian window and discrete Fourier transform. The mean frequency spectrum over cycles can be further computed. The spectrum magnitudes from 30 Hz to 790 Hz with 10 Hz interval are taken as features. The maximum frequency 790 Hz is considered to adapt possible murmurs. So, 77 features for S1 state are obtained. Similar operation is done to S2, systole, and diastole state. So, the total number of features obtained from frequency domain for states is 77 features × 4 = 308 features.

#### 2.5.3. Normalized Amplitude Features (12 Features)

Previous physiological findings in amplitude of heart sound [1–3] disclosed that the amplitude is related to heart hemodynamics. So, it is reasonable to extract features from amplitude of heart sounds. To eliminate the difference between subjects and records, no absolute amplitude is considered. Relative ratios of amplitude between states are extracted as given in Table 2.

#### 2.5.4. Energy-Domain Features (47 Features)

The features in energy domain consist of two parts: the energy ratio of a band-pass signal to the original one and the energy ratio of one state to another.

For the first part, various frequency bands are considered with initial value of 10 Hz and increment bandwidth of 30 Hz; i.e., the 27 frequency bands are [10  40] Hz, [40  70] Hz, [70  100] Hz,…, and [790  820] Hz, respectively. The previous studies disclosed that murmurs' frequency is hardly higher than 800 Hz. In order to reflect murmurs' properties, the maximum frequency considered in this domain is 820 Hz. In this paper, each band-pass filter is designed by a five-order Butterworth filter. The output of the* i*th filter is **y**_*i*_:

(1) $$\begin{array}{c} y_{i}=filter\left(\;b_{i},a_{i},x\;\right),\;i=1,\ldots ,27, \end{array}$$

where **b**_*i*_ (numerator) and **a**_*i*_ (denominator) are the Butterworth IIR filter coefficient vectors. Hence, the energy ratio is defined as

(2) $$\begin{array}{c} Ratio\_band\_energy_{i}=\frac{\sum {\left|y_{i}\right|}^{2}}{\sum {\left|x\right|}^{2}},\;i=1,\ldots ,27. \end{array}$$

It is known that a normal heart sound signal generally has a frequency band blow 200 Hz. However, the frequency band may extend to 800 Hz if it contains murmurs. So, the energy ratio reflects signal energy distribution along frequency band. These features are helpful to discriminate a PCG records with murmurs or not.

For the second part, the relative energy ratio is investigated between any two states, resulting in another 20 features. The energy ratio of S1 to the cycle period is defined as

(3) Ratio_state_energyS1_cycle=∑ns1in2∑ncin2,i=1,…,N,

where* N* is the number of cycles in a PCG recording and *n* is the discrete time index. The authors consider average of *Ratio*_*state*_*energy*_*S*1_*cycle*_ and standard deviation of *Ratio*_*state*_*energy*_*S*1_*cycle*_ as two features. Similarly, another 18 features can be obtained from the averages and the standard deviations. The 47 proposed features in this domain are listed in Table 3.

#### 2.5.5. Spectrum-Domain for Records Features (27 Features)

As is mentioned in Section 2.5.4, the frequency band is divided into 27 bands with start from 10 Hz to 30 Hz increment. Fast Fourier transform is performed for every record. The ratio of spectrum magnitude sum in a band to spectrum magnitude sum in whole band is taken as a feature. So, 27 features can be produced for a record. These features can discriminate murmurs because murmurs generally have higher frequency than those of normal heart sounds.

#### 2.5.6. Cepstrum-Domain Features (13 Features × 5 = 65 Features)

The cepstrum of a PCG recording is calculated and the first 13 cepstral coefficients are taken as features [36]. Additionally, all S1 states from a PCG recording are joined together to create a new digital sequence. Then, the cepstrum can be calculated and the first 13 cepstral coefficients are taken as features. Similarly, the same operation is done to S2, systole, and diastole states. So, another 13 features × 3 = 39 features are obtained. The cepstrum of a signal *p*(*n*) is computed as follows:

(4) Pk=DFTpn,

(5) P^k=log⁡Pk,

(6) p^n=IDFTP^k,

where the operator DFT[.] is the discrete Fourier transform, IDFT[.] is the inverse DFT, log⁡[.] is the natural logarithm, and |.| is the absolute operation. It is known that the cepstrum coefficient decays quickly. So, it is reasonable to select the first 13 coefficients as features. The cepstrum-domain features are listed in Table 4.

#### 2.5.7. Cyclostationary Features (4 Features)

**Figure 3:**
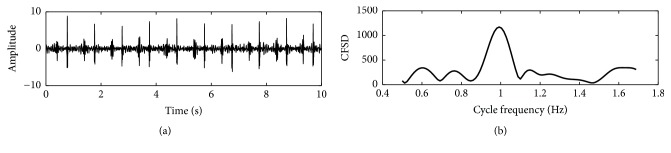
An example of cycle frequency spectral density. (a) A subsequence of a PCG recording and (b) cycle frequency spectral density of the subsequence.

(1) m_cyclostationarity_1 is mean value of the degree of cyclostationarity. The definition of “degree of cyclostationarity” can be found in [37]. This feature indicates the degree of a signal repetition. It will be infinite if the events which occurred in heart beating were exactly periodic. However, it will be a small number if the events are randomly alike. Let us assume *γ*(*α*) is the cycle frequency spectral density (CFSD) of a heart sound signal at cycle frequency *α*, as shown in Figure 3. This feature is defined as

(7) $$\begin{array}{c} d\left(\;\eta \;\right)=\frac{\gamma \left(\eta \right)}{\int_{0}^{\beta }\gamma \left(\alpha \right)d\alpha }, \end{array}$$

where *β* is the maximum cycle frequency considered and *η* is the basic cycle frequency indicated by the main peak location of *γ*(*α*). A heart sound signal is equally divided into subsequences. The feature can be estimated for each subsequence; then the mean value and standard deviation can be obtained.

(2) sd_cyclostationarity_1 is SD of the degree of cyclostationarity.

(3) m_cyclostationarity_2 is mean value of the sharpness measure. The definition of this indicator is the sharpness of the peak of cycle frequency spectral density. It is

(8) $$\begin{array}{c} peak\_sharpness=\frac{\max\left(\gamma \left(\alpha \right)\right)}{median\left(\gamma \left(\alpha \right)\right)}. \end{array}$$

The operators max(.) and median(.) are the maximum and median magnitude of the cycle frequency spectral density. It is obvious that the sharper the peak is, the greater the feature is. Similarly, the feature can be calculated for each subsequence of the heart sound signal and then get the mean value and SD.

(4) sd_cyclostationarity_2 is SD of the sharpness measure.

The four features are listed in Table 5.

#### 2.5.8. High-Order Statistics Features (16 Features)

In probability theory and statistics, skewness is a measure of the asymmetry of the probability distribution of real-valued random numbers about its mean. It is a three-order statistics. Kurtosis is a measure of “tailedness” of the probability distribution of real-valued random numbers. It is a four-order statistics. The skewness and kurtosis of each state are considered here. There are sixteen related features, as listed in Table 6.

#### 2.5.9. Entropy Features (16 Features)

Sample entropy (SampEn) and fuzzy measure entropy (FuzzyMEn) have the ability to measure the complexity of a random sequence [38, 39]. Sample entropy and fuzzy measure entropy are both computed to measure the complexity of every state segmented by Springer's algorithm. Then, the average and standard deviation are used as the features. The detailed algorithm to calculate sample entropy and fuzzy measure entropy can be found in [38, 39]. So, 16 features in entropy are listed in Table 7.

#### 2.5.10. Summary

This paper considers 515 features in nine domains. They are listed in Table 8 for reference. To the authors' knowledge, the features extracted from entropy and cyclostationarity are new for heart sound classification. On the other hand, the combination of the features in the nine domains is novel for this classification. Seldom previous study has considered so many features simultaneously.

### 2.6. SVM-Based Model for Signal Quality Estimation and Classification

The signal quality classification is typically two-category classification problem in this study. The SVM-based model has yielded excellent results in many two-class classification situations. Given a training sample set {**x**_*i*_, *y*_*i*_}, *i* = 1, ⋯, *K*, where **x**_*i*_ is the feature vector **x**_*i*_ ∈ *R*^*d*^, *y*_*i*_ is the label. So, SVM-based model is applicable for both signal quality estimation and classification. For the quality estimation, the label is *y*_*i*_ ∈ {1,0}, which means good quality and bad quality. For the classification, the label is *y*_*i*_ ∈ {1, −1}, which means abnormal and normal cases. The aim of SVM classifier is to develop optimal hyperplane between two classes besides distinguishing them. The optimal hyperplane can also be constructed by calculating the following optimization problem.

(9) min ϕw=12wTw+C∑i=1Kξisubject  to yiwTφxi+b≥1,i=1,⋯,K.

Here *ξ*_*i*_ is a relaxation variable and *ξ*_*i*_ ≥ 0, *C* is a penalty factor, and **w** is the coefficient vector. *φ*(**x**_*i*_) is introduced to get a nonlinear support vector machine. The optimization problem can be equally transformed into

(10) max Lα=∑i=1Kαi−12∑i,j=1Kαiαjyiyjκxi,xjsubject  to ∑i=1Kαiyi=0,0≤αi≤C,  i=1,⋯,K

where *κ*(**x**_*i*_, *y*_*i*_) is a kernel function. The authors use RBF kernel function in this paper. And the parameter sigma is empirically set as 14. The discussions about the selection of kernel function and the influence of sigma are given in the Section 4.3.

### 2.7. Scoring

The overall score is computed based on the number of records classified as normal, uncertain, or abnormal, in each of the reference categories. These numbers are denoted by *Nn*_*k*_, *Nq*_*k*_, *Na*_*k*_, *An*_*k*_, *Aq*_*k*_, and *Aa*_*k*_ in Table 9.

Weights for the various categories are defined as follows (based on the distribution of the complete test set):

(11) wa1=clean  abnormal  recordstotal  abnormal  records,

(12) wa2=noisy  abnormal  recordstotal  abnormal  records.

(13) wn1=clean  normal  recordstotal  normal  records,

(14) wn2=noisy  normal  recordstotal  normal  records.

The modified sensitivity and specificity are defined as (based on a subset of the test set)

(15) Se=wa1Aa1Aa1+Aq1+An1+wa2Aa2+Aq2Aa2+Aq2+An2,

(16) Sp=wn1Nn1Na1+Nq1+Nn1+wn2Nn2+Nq2Na2+Nq2+Nn2.

The overall score is then the average of these two values:

(17) $$\begin{array}{c} \text{Overall score}=\frac{\left(Se+Sp\right)}{2}. \end{array}$$

## 3. Results

### 3.1. Correlation Analysis between the Features and the Target Label

**Figure 4:**
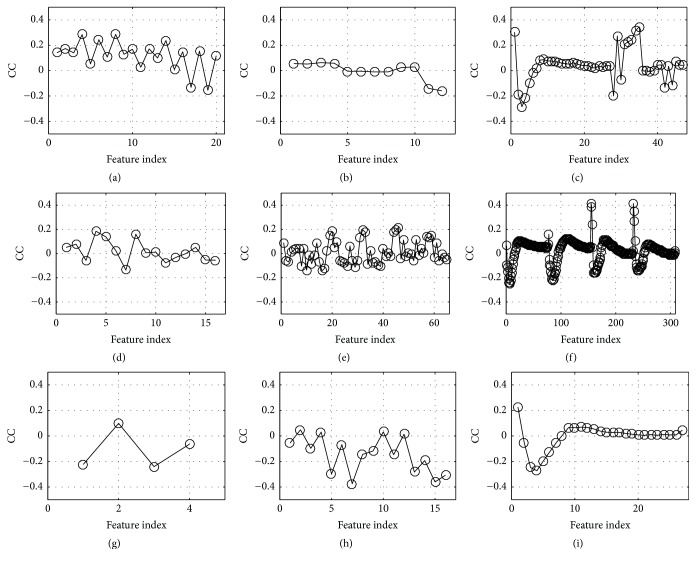
Correlation coefficient (CC) between features and the target label. (a) Time interval, (b) state amplitude, (c) energy, (d) high-order statistics, (e) cepstrum, (f) frequency spectrum of state, (g) cyclostationarity, (h) entropy, and (i) frequency spectrum of records.

In this paper, a total of 515 features are extracted from a single recording. A question arises about how to evaluate the contribution of a feature for classification. To answer the question, correlation analysis is performed between the features and the target label. The correlation coefficients are plotted in the nine domains in Figure 4. The statistics of the coefficients are listed in Table 10. The top coefficient is 0.417 which is from “frequency spectrum of state” at 30 Hz of S2 state. This feature is called “top feature”. The statistics of top features are listed in Table 11. It is shown that 4 in the top 10 features are from “frequency spectrum of state”. So, this domain is ranked the first. Both “energy” and “entropy” contribute 3 in top 10. But “energy” contributes 12 in top 100 which is greater than “entropy” who contributes 8 in top 100. Therefore, “energy” is ranked the 2nd and “entropy” is ranked the 3rd. Following similar logics, the nine domains are ranked as shown in Table 11. It concludes that the domain “frequency spectrum of state” contributes the most. “Energy” and “entropy” are the second and third place to contribute.

### 3.2. Signal Quality Estimation by SVM

The SVM model in (9) is used to discriminate signal quality. The reference labels for clean and noisy PCG recordings are “1” and “0”, respectively. The input to the model is the proposed 515 features. The performance is tested by various input, as shown in Table 12. Firstly, 10% of randomly selected data are used for training and the other 90% of data are used for testing without any overlap. Then the percent of train data increases by 10% and repeats. The performance summary for signal quality estimation is listed in Table 12. The manual reference indicates that there are 2874 clean recordings and 279 noisy recordings. So, the numbers of the two quality groups have great unbalance which has bad effect on network training. It is shown that the performance for good signal quality has excellent sensitivity from 96% to 98% no matter how much the percent of data for training varies from 10% to 90%. However, the performance for bad signal quality is poor. The specificity is around 50%; even the training data varies from 10% to 90%. Fortunately, this performance has little influence on the final classification, shown in the next subsection.

### 3.3. Classification of Normal/Abnormal by SVM

The classification of normal/abnormal is carried out by the SVM model as given in (9). The 515-feature vector is used as input to the SVM network and the label is used as output. The SVM model is firstly trained by a part of data and then tested by the other. To test the generation ability of the model, it is widely tested in following two cases.

**Case 1 .**

All data (3153 recordings) are used to train the model and all data (3153 recordings) are to test the model. So, the training data and the testing data are fully overlapped.

**Case 2 .**

10% of the normal recordings and 10% of the abnormal recordings are randomly selected to train the model, and the other 90% are to test the model. The training data and the testing data are exclusively nonoverlapped. This program independently repeats 200 times to evaluate the stability. Sensitivity and specificity are calculated in “mean±SD” to indicate the classification performance. Then, the percent of training data increases by 10%, the percent of test data decreases by 10%, and the evaluation process is repeated until the percent of training data reaches 90%.

The performance of the proposed classification is listed in Table 13. The overall score of Case 1 is up to 0.95. It proves that the proposed features are effective for this classification. In Case 2, it can be found that, with the increasing percent of data for training, both sensitivity and specificity increase. The standard deviation is not greater than 0.02. So, the score variation is very small; even the classifier independently runs 200 times. This simulation proves that the proposed features and the model have excellent generation ability and stability and are effective in discriminating the PCG recordings.

## 4. Discussions

### 4.1. Effect of the Number of Top Features

**Figure 5:**
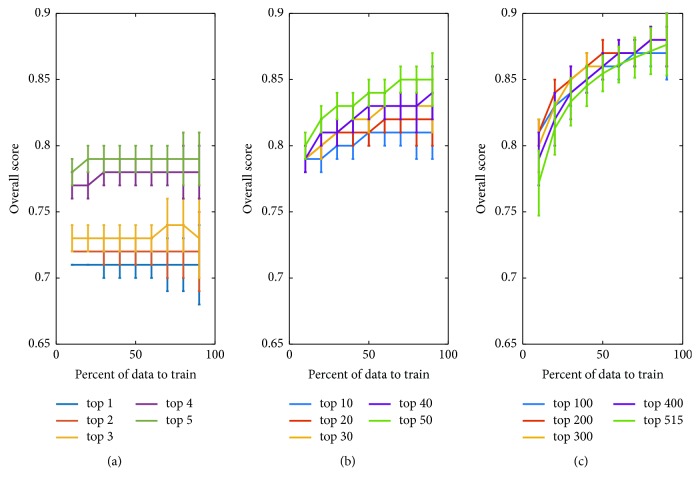
Classification performance with respect to the number of top features and percent of data for training. (a) Overall scores obtained by top 1, top 2, top 3, top 4, and top 5 features. (b) Overall scores obtained by top 10, top 20, top 30, top 40, and top 50 features. (c) Overall scores obtained by top 100, top 200, top 300, top 400, and top 515 features.

This paper proposes 515 features from multidomains. However, correlation analysis shows that each feature has different degree of correlation with the target label. The performance will change with the number of selected features. To evaluate the effect of selected features, the authors conduct simulations under condition of varying the top number of features. The mean overall score changing with respect to the number of top features is illustrated in Figure 5, where Figure 5(a) shows the performance with top 1 to top 5 features, Figure 5(b) is with top 10 to top 50 features, and Figure 5(c) is with top 100 to top 515 features. It can be seen that there are two factors to influence SVM classifier's generalization ability. One is the percent of data for training; the other is the number of top features. An overall look shows that both the two factors have positive effect on the classification performance. Roughly speaking, if any one of them increases, the performance will get improvement. However, it is not always true. A closer look at Figure 5(a) indicates the performance has little change as the percent increases. But the performance gets much improvement as the percent increases, shown in Figures 5(b) and 5(c), where the number of top features is much greater than that in Figure 5(a). A careful look at Figure 5(c) discloses that the case that all the features (515) are involved does not result in the best performance. It can be found that there is an “edge effect” on the selection of top feature number. That is, much improvement can be obtained via increasing top feature number as the number is small. However, the improvement becomes little when the number is up to some degree. The best performance is obtained with top 400 features in this paper. The performance will get worse if the number continues to increase.

The proposed classification has very good performance even if the number of features is small. For example, it can be noted in Figure 5(a) that the overall score is up to 0.71 as only the top 1 feature is used and the score increases to 0.81 when the top 10 features are used. This is one of attractive advantages of the proposed classification.

Another advantage is that the proposed SVM classifier has very stable output. Even if the SVM classifier is trained independently by randomly selected features, the overall score has very low variations (standard variance is approximately lower than 0.02). That is to say, the proposed features and SVM classifier are adaptable to the classification.

### 4.2. Classification Performance Based on Features in Specified Domain

Table 13 and Figure 5 show the classification performance based on mixed features from multidomains. It is interesting to test the performance based on features of a separated domain. This test would be evidence to show the power of combined features for classification. So, the SVM classifier and 10-fold validation are used for this purpose. The results are listed in Table 14. It is seen that, the highest score, 0.85, is produced if only the features in “frequency spectrum of state” are used. Other high scores are obtained based on features in domain of “entropy”, “energy”, and “cepstrum”. It can be found that these results are almost coincident with those of correlation analysis given in Section 3.1 where “frequency spectrum of state”, “energy”, “entropy”, and “cepstrum” are the top domains to contribute effective features. This simulation indicates that it is reasonable to improve classification performance by combining multidomain features.

### 4.3. Selection of Kernel Function and Influence of the Gaussian Kernel Function

Typically, the kernel functions for a SVM have several selections, such as linear kernel, polynomial kernel, sigmoid kernel, and Gaussian radial basis function. Given an arbitrary dataset, one does not know which kernel may work best. Generally, one can start with the simplest hypothesis space first and then work a way up towards a more complex hypothesis space. The authors followed this lesson summarized by the previous researchers. A bad performance was produced by the SVM classifier using a linear kernel since 515 features in this study were complex and they were not linearly separable, as well as using a polynomial kernel. The authors actually tried out all possible kernels and found that the RBF kernel produced the best performance. The authors believed that the best performance should be attributed to the RBF kernel's advantages. The first is translation invariance. Let the RBF kernel be *K*(**x**_*i*_, **x**_*j*_) = exp⁡(−‖**x**_*i*_ − **x**_*j*_‖/*γ*); then *K*(**x**_*i*_, **x**_*j*_) = *K*(**x**_*i*_ + ***ν***, **x**_*j*_ + ***ν***) where ***ν*** is any arbitrary vector. It is known that the kernel is effectively a similarity measure. The invariance is useful to measure the similarity between the proposed features. The second is that the similarity is measured by Euclidean distance. RBF kernel is a function of the Euclidean distance between the features. In this study, the Euclidean distance is a preferred similarity metric. The authors selected the RBF kernel function because the advantages match the classification purpose and features.

**Figure 6:**
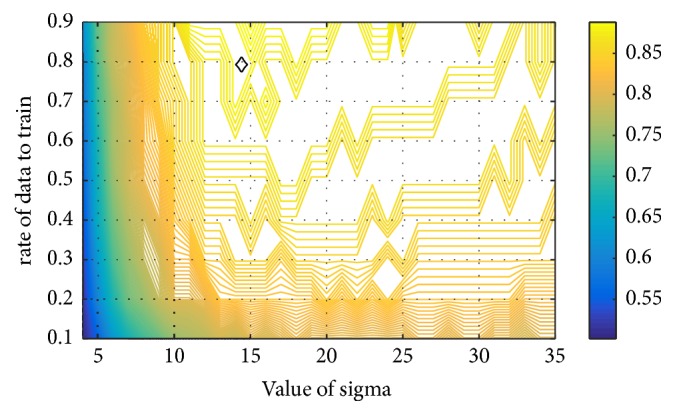
The mean overall score with respect to rate of data for training and value of sigma. The diamond shows the peak position.

One difficulty with the Gaussian RBF kernel function is the parameter sigma governing the kernel width. A general conclusion about sigma has been summarized by previous researchers. A large value of sigma may lead to an over smoothing hyperplane and a washing out of structure that might otherwise be extracted from the feature space. A reducing value of sigma may lead to a noisy hyperplane elsewhere in the feature space where the feature density is smaller. There is a trade-off between sensitivity to noise at small value and over smoothing at large value. To select appropriate value for sigma, the authors did grid search in a specified range, as seen in Figure 6. This figure shows the mean overall score, based on 50 independent runs, with respect to rate of data for training and value of sigma where the value of sigma increases from 4 to 35 by step of 1 and the rate of data for training increases from 0.1 to 0.9 by step of 0.1. It is found that the peak of the overall score occurs at sigma 14 as indicated by the diamond.

## 5. Conclusions

In this paper, 515 features are extracted from multiple domains, i.e., time interval, state amplitude, energy, high-order statistics, cepstrum, frequency spectrum, cyclostationarity, and entropy. Correlation analysis between the features and the target label shows that the features from frequency spectrum contribute the most to the classification. The features and the SVM classifier jointly show the powerful classification performance. The results show the overall score reaches 0.88±0.02 based on 200 independent simulations, which is competitive to the previous best classification methods. Moreover, the SVM classifier has very good performance with even small number of features for training and has stable output regardless of randomly selected features for training.

## Figures and Tables

**Table 1:** Summary of time-domain features.

Feature index	Feature name	Physical meaning
1	m_RR	mean value of RR intervals
2	sd_RR	standard deviation (SD) of RR intervals
3	m_IntS1	mean value of S1 intervals
4	sd_IntS1	SD of S1 intervals
5	m_IntS2	mean value of S2 intervals
6	sd_IntS2	SD of S2 intervals
7	m_IntSys	mean value of systolic intervals
8	sd_IntSys	SD of systolic intervals
9	m_IntDia	mean value of diastolic intervals
10	sd_IntDia	SD of diastolic intervals
11	m_Ratio_SysRR	mean value of the ratio of systolic interval to RR interval of each heart beat
12	sd_Ratio_SysRR	SD of the ratio of systolic interval to RR interval of each heart beat
13	m_Ratio_DiaRR	mean value of the ratio of diastolic interval to RR interval of each heart beat
14	sd_Ratio_DiaRR	SD of the ratio of diastolic interval to RR interval of each heart beat
15	m_Ratio_SysDia	mean value of the ratio of systolic to diastolic interval of each heart beat
16	sd_Ratio_SysDia	SD of the ratio of systolic to diastolic interval of each heart beat
17	m_Ratio_S1RR*∗*	mean value of the ratio of S1 interval to RR interval of each heart beat
18	sd_Ratio_S1RR*∗*	SD of the ratio of S1 interval to RR interval of each heart beat
19	m_Ratio_S2RR*∗*	mean value of the ratio of S2 interval to RR interval of each heart beat
20	sd_Ratio_S2RR*∗*	SD of the ratio of S2 interval to RR interval of each heart beat

Note: *∗* means the new added features in this study.

**Table 2:** Summary of normalized amplitude features.

Feature index	Feature name	Physical meaning
1	m_Amp_SysS1	mean value of the ratio of the mean absolute amplitude during systole to that during S1 in each heart beat
2	sd_Amp_SysS1	SD of m_Amp_SysS1
3	m_Amp_DiaS2	mean value of the ratio of the mean absolute amplitude during diastole to that during S2 in each heart beat
4	sd_Amp_DiaS2	SD of m_Amp_DiaS2
5	m_Amp_S1S2	mean value of the ratio of the mean absolute amplitude during S1 to that during S2 in each heart beat
6	sd_Amp_S1S2	SD of m_Amp_S1S2
7	m_Amp_S1Dia	mean value of the ratio of the mean absolute amplitude during S1 to that during diastole in each heart beat
8	sd_Amp_S1Dia	SD of m_Amp_S1Dia
9	m_Amp_SysDia	mean value of the ratio of the mean absolute amplitude during systole to that during diastole in each heart beat
10	sd_Amp_SysDia	SD of m_Amp_SysDia
11	m_Amp_S2Sys	mean value of the ratio of the mean absolute amplitude during S2 to that during systole in each heart beat
12	sd_Amp_S2Sys	SD of m_Amp_S2Sys

**Table 3:** Summary of energy-domain features.

Feature index	Feature name	Physical meaning
1-27	*Ratio*_*band*_*energy*_*i*_	Ratio of a given band energy to the total energy
28	*m*_*Ratio*_*state*_*energy*_*S*1_*cycle*_	Mean of *Ratio*_*state*_*energy*_*S*1_*cycle*_
29	*SD*_*Ratio*_*state*_*energy*_*S*1_*cycle*_	standard deviation of *Ratio*_*state*_*energy*_*S*1_*cycle*_
30	*m*_*Ratio*_*state*_*energy*_*S*2_*cycle*_	Mean of *Ratio*_*state*_*energy*_*S*2_*cycle*_
31	*SD*_*Ratio*_*state*_*energy*_*S*2_*cycle*_	standard deviation of *Ratio*_*state*_*energy*_*S*2_*cycle*_
32	*m*_*Ratio*_*state*_*energy*_*sys*_*cycle*_	Mean of *Ratio*_*state*_*energy*_*sys*_*cycle*_
33	*SD*_*Ratio*_*state*_*energy*_*sys*_*cycle*_	standard deviation of *Ratio*_*state*_*energy*_*sys*_*cycle*_
34	*m*_*Ratio*_*state*_*energy*_*dia*_*cycle*_	Mean of *Ratio*_*state*_*energy*_*dia*_*cycle*_
35	*SD*_*Ratio*_*state*_*energy*_*dia*_*cycle*_	standard deviation of *Ratio*_*state*_*energy*_*dia*_*cycle*_
36	*m*_*Ratio*_*state*_*energy*_*S*1_*S*2_	Mean of *Ratio*_*state*_*energy*_*S*1_*S*2_
37	*SD*_*Ratio*_*state*_*energy*_*S*1_*S*2_	standard deviation of *Ratio*_*state*_*energy*_*S*1_*S*2_
38	*m*_*Ratio*_*state*_*energy*_*S*1_*sys*_	Mean of *Ratio*_*state*_*energy*_*S*1_*sys*_
39	*SD*_*Ratio*_*state*_*energy*_*S*1_*sys*_	standard deviation of *Ratio*_*state*_*energy*_*S*1_*sys*_
40	*m*_*Ratio*_*state*_*energy*_*S*1_*dia*_	Mean of *Ratio*_*state*_*energy*_*S*1_*dia*_
41	*SD*_*Ratio*_*state*_*energy*_*S*1_*dia*_	standard deviation of *Ratio*_*state*_*energy*_*S*1_*dia*_
42	*m*_*Ratio*_*state*_*energy*_*S*2_*sys*_	Mean of *Ratio*_*state*_*energy*_*S*2_*sys*_
43	*SD*_*Ratio*_*state*_*energy*_*S*2_*sys*_	standard deviation of *Ratio*_*state*_*energy*_*S*2_*sys*_
44	*m*_*Ratio*_*state*_*energy*_*S*2_*dia*_	Mean of *Ratio*_*state*_*energy*_*S*2_*dia*_
45	*SD*_*Ratio*_*state*_*energy*_*S*2_*dia*_	standard deviation of *Ratio*_*state*_*energy*_*S*2_*dia*_
46	*m*_*Ratio*_*state*_*energy*_*dia*_*sys*_	Mean of *Ratio*_*state*_*energy*_*dia*_*sys*_
47	*SD*_*Ratio*_*state*_*energy*_*dia*_*sys*_	standard deviation of *Ratio*_*state*_*energy*_*dia*_*sys*_

**Table 4:** Summary of cepstrum-domain features.

Feature index	Feature name	Physical meaning
1-13	Cepstrum coefficients	Cepstrum coefficients of a PCG recording
14-26	Cepstrum coefficients	Cepstrum coefficients of jointed S1 state
27-39	Cepstrum coefficients	Cepstrum coefficients of jointed systolic state
40-52	Cepstrum coefficients	Cepstrum coefficients of jointed S2 state
53-65	Cepstrum coefficients	Cepstrum coefficients of jointed diastole state

**Table 5:** Summary of cyclostationary features.

Feature index	Feature name	Physical Meaning
1	m_cyclostationarity_1	mean value of the degree of cyclostationarity
2	sd_cyclostationarity_1	SD of the degree of cyclostationarity
3	m_cyclostationarity_2	mean value of the sharpness measure
4	sd_cyclostationarity_2	SD of the sharpness measure

**Table 6:** Summary of high-order statistics features.

Feature index	Feature name	Physical Meaning
1	m_S1_skewness	mean value of the skewness of S1
2	sd_S1_skewness	SD of the skewness of S1
3	m_S1_kurtosis	mean value of the kurtosis of S1
4	sd_S1_kurtosis	SD of the kurtosis of S1
5	m_S2_skewness	mean value of the skewness of S2
6	sd_S2_skewness	SD of the skewness of S2
7	m_S2_kurtosis	mean value of the kurtosis of S2
8	sd_S2_kurtosis	SD of the kurtosis of S2
9	m_sys_skewness	mean value of the skewness of systole
10	sd_sys_skewness	SD of the skewness of systole
11	m_sys_kurtosis	mean value of the kurtosis of systole
12	sd_sys_kurtosis	SD of the kurtosis of systole
13	m_dia_skewness	mean value of the skewness of diastole
14	sd_dia_skewness	SD of the skewness of diastole
15	m_dia_kurtosis	mean value of the kurtosis of diastole
16	sd_dia_kurtosis	SD of the kurtosis of diastole

**Table 7:** Summary of entropy features.

Feature index	Feature name	Physical meaning
1	*m*_*SampEn*_*S*1	Mean value of SampEn of S1 state
2	*SD*_*SampEn*_*S*1	SD value of SampEn of S1 state
3	*m*_*SampEn*_*S*2	Mean value of SampEn of S2 state
4	*SD*_*SampEn*_*S*2	SD value of SampEn of S2 state
5	*m*_*SampEn*_*sys*	Mean value of SampEn of systolic state
6	*SD*_*SampEn*_*sys*	SD value of SampEn of systolic state
7	*m*_*SampEn*_*dia*	Mean value of SampEn of diastolic state
8	*SD*_*SampEn*_*dia*	SD value of SampEn of diastolic state
9	*m*_*FuzzyMEn*_*S*1	Mean value of FuzzyMEn of S1 state
10	*SD*_*FuzzyMEn*_*S*1	SD value of FuzzyMEn of S1 state
11	*m*_*FuzzyMEn*_*S*2	Mean value of FuzzyMEn of S2 state
12	*SD*_*FuzzyMEn*_*S*2	SD value of FuzzyMEn of S2 state
13	*m*_*FuzzyMEn*_*sys*	Mean value of FuzzyMEn of systolic state
14	*SD*_*FuzzyMEn*_*sys*	SD value of FuzzyMEn of systolic state
15	*m*_*FuzzyMEn*_*dia*	Mean value of FuzzyMEn of diastolic state
16	*SD*_*FuzzyMEn*_*dia*	SD value of FuzzyMEn of diastolic state

**Table 8:** Summary of the proposed features.

Index	Domain	Num. of features	Motivation
1	Time interval	20	The time interval of each state has physiological meaning based on heart physiology.
2	Frequency spectrum of state	308	To reflect the frequency spectrum within state.
3	State amplitude	12	The amplitude is related to the heart hemodynamics.
4	Energy	47	To reflect energy distribution with respect to frequency band
5	Frequency spectrum of records	27	To reflect frequency spectrum within records
6	Cepstrum	65	To reflect the acoustic properties.
7	Cyclostationary	4	To reflect the degree of signal repetition.
8	High-order statistics	16	To reflect the skewness and kurtosis of each signal state.
9	Entropy	16	To reflect the PCG signal inherent complexity.
Total	-* *-	515	-* *-

**Table 9:** Variables to evaluate the classification.

		Classification Results		
		Normal (-1)	Uncertain (0)	Abnormal (1)
Reference label	Normal, clean	*Nn*_1_	*Nq*_1_	*Na*_1_
	Normal, noisy	*Nn*_2_	*Nq*_2_	*Na*_2_
	Abnormal, clean	*An*_1_	*Aq*_1_	*Aa*_1_
	Abnormal, noisy	*An*_2_	*Aq*_2_	*Aa*_2_

**Table 10:** Summary of the correlation coefficients.

No.	Feature domain	Max. absolute CC	Physical meaning
1	Time interval	0.286	sd_IntSys
2	State amplitude	-0.159	sd_Amp_S2Sys
3	Energy	0.345	Standard deviation of *Ratio*_*state*_*energy*_*dia*_*cycle*_
4	High-order statistics	0.185	sd_S1_kurtosis
5	Cepstrum	0.216	The seventh cepstrum coefficient of S2 state
6	Frequency spectrum of state	0.417	Spectrum value of 30 Hz of S2 state
7	cyclostationarity	-0.240	Sharpness of the peak of cycle frequency spectral density
8	Entropy	-0.374	Average value of sample entropy of diastolic state
9	Frequency spectrum of records	-0.272	Ratio of spectrum magnitude sum in [90 120] Hz

**Table 11:** Rank order of the nine domains based on contribution.

Rank order	Feature domain (Total num. of features)	Num. of top 10 features	Num. of top 100 features	Num. of top 200 features	Num. of top 300 features
1	Frequency spectrum of state (308)	4	39	115	183
2	Energy (47)	3	12	16	24
3	Entropy (16)	3	8	10	11
4	Cepstrum (65)	0	14	28	40
5	Time interval (20)	0	14	17	17
6	Frequency spectrum of records (27)	0	5	5	10
7	High-order statistics (16)	0	4	4	7
8	Cyclostationarity (4)	0	2	3	4
9	State amplitude (12)	0	2	2	4

**Table 12:** Performance of signal quality classification.

Percent data to train	Percent data to test	Estimation results for Reference, clean		Estimation results for Reference, noisy		Sensitivity	Specificity
		Clean	Noisy	Clean	Noisy		

10%	90%	2421	166	104	146	0.96	0.47
20%	80%	2143	154	75	149	0.97	0.49
30%	70%	1869	143	59	136	0.97	0.49
40%	60%	1601	125	44	122	0.97	0.49
50%	50%	1332	104	36	104	0.97	0.50
60%	40%	1064	84	28	84	0.97	0.50
70%	30%	799	63	19	64	0.97	0.50
80%	20%	530	44	12	45	0.98	0.50
90%	10%	265	21	6	22	0.98	0.50

**Table 13:** Performance of the classification.

Case	Percent of data to train	Percent of data to test	Repeat times	Training and test data division	Sensitivity	Specificity	Overall score
Case 1	100%	100%	1	No	0.99	0.91	0.95

Case 2	10%	90%	200	Yes	0.68±0.06	0.87±0.03	0.77±0.02
	20%	80%	200	Yes	0.76±0.05	0.86±0.02	0.81±0.02
	30%	70%	200	Yes	0.80±0.04	0.87±0.02	0.83±0.02
	40%	60%	200	Yes	0.82±0.04	0.87±0.01	0.85±0.02
	50%	50%	200	Yes	0.84±0.03	0.87±0.01	0.85±0.01
	60%	40%	200	Yes	0.85±0.04	0.87±0.01	0.86±0.01
	70%	30%	200	Yes	0.86±0.04	0.87±0.01	0.87±0.02
	80%	20%	200	Yes	0.87±0.04	0.87±0.02	0.87±0.02
	90%	10%	200	Yes	0.88±0.04	0.87±0.02	0.88±0.02

Note: the number is presented as mean±SD.

**Table 14:** Classification performance based on features in specified domain.

Rank	Domain (# features)	Mean of overall score	Standard deviation
1	Frequency spectrum of state (308)	0.85	0.021
2	Entropy (16)	0.82	0.028
3	Energy (47)	0.78	0.020
4	Cepstrum (65)	0.75	0.027
5	High-order statistics (16)	0.73	0.029
6	Frequency spectrum of records (27)	0.71	0.025
7	Time interval (20)	0.70	0.025
8	Cyclostationarity (4)	0.65	0.042
9	State amplitude (12)	0.61	0.025

## Data Availability

The authors state that the data used in this study are available from the website http://www.physionet.org/challenge/2016/ to support the conclusions.

## References

[1] Luisada A. A., Liu C. K., Aravanis C., Testelli M., Morris J. (1958). On the mechanism of production of the heart sounds. *American Heart Journal *.

[2] Sakamoto T., Kusukawa R., Maccanon D. M., Luisada A. A. (1965). Hemodynamic determinants of the amplitude of the first heart sound. *Circulation Research*.

[3] Sakamoto T., Kusukawa R., MacCanon D. M., Luisada A. A. (1966). First heart sound amplitude in experimentally induced alternans. *CHEST*.

[4] Durand L.-G., Pibarot P. (1995). Digital signal processing of the phonocardiogram: Review of the most recent advancements. *Critical Reviews in Biomedical Engineering*.

[5] Gerbarg D. S., Taranta A., Spagnuolo M., Hofler J. J. (1963). Computer analysis of phonocardiograms. *Progress in Cardiovascular Diseases*.

[6] Akay Y., Akay M., Welkowitz W., Kostis J. (1994). Noninvasive detection of coronary artery disease. *IEEE Engineering in Medicine and Biology Magazine*.

[7] Durand L.-G., Blanchard M., Cloutier G., Sabbah H. N., Stein P. D. (1990). Comparison of Pattern Recognition Methods for Computer-Assisted Classification of Spectra of Heart Sounds in Patients with a Porcine Bioprosthetic Valve Implanted in the Mitral Position. *IEEE Transactions on Biomedical Engineering*.

[8] Uğuz H. (2012). A biomedical system based on artificial neural network and principal component analysis for diagnosis of the heart valve diseases. *Journal of Medical Systems*.

[9] Ölmez T., Dokur Z. (2003). Classification of heart sounds using an artificial neural network. *Pattern Recognition Letters*.

[10] Dokur Z., Ölmez T. (2008). Heart sound classification using wavelet transform and incremental self-organizing map. *Digital Signal Processing*.

[11] Ari S., Hembram K., Saha G. (2010). Detection of cardiac abnormality from PCG signal using LMS based least square SVM classifier. *Expert Systems with Applications*.

[12] Maglogiannis I., Loukis E., Zafiropoulos E., Stasis A. (2009). Support Vectors Machine-based identification of heart valve diseases using heart sounds. *Computer Methods and Programs in Biomedicine*.

[13] Zheng Y., Guo X., Ding X. (2015). A novel hybrid energy fraction and entropy-based approach for systolic heart murmurs identification. *Expert Systems with Applications*.

[14] Bentley P. M., Grant P. M., McDonnell J. T. E. (1998). Time-frequency and time-scale techniques for the classification of native and bioprosthetic heart valve sounds. *IEEE Transactions on Biomedical Engineering*.

[15] Avendaño-Valencia L. D., Godino-Llorente J. I., Blanco-Velasco M., Castellanos-Dominguez G. (2010). Feature extraction from parametric time-frequency representations for heart murmur detection. *Annals of Biomedical Engineering*.

[16] Quiceno-Manrique A. F., Godino-Llorente J. I., Blanco-Velasco M., Castellanos-Dominguez G. (2010). Selection of dynamic features based on time-frequency representations for heart murmur detection from phonocardiographic signals. *Annals of Biomedical Engineering*.

[17] Yang T., Hsieh H. Classification of Acoustic Physiological Signals Based on Deep Learning Neural Networks with Augmented Features.

[18] Tschannen M., Kramer T., Marti G., Heinzmann M., Wiatowski T. Heart sound classification using deep structured features.

[19] Nilanon T., Yao J., Hao J., Purushotham S., Liu Y. Normal / abnormal heart sound recordings classification using convolutional neural network.

[20] Diaz Bobillo I. A Tensor Approach to Heart Sound Classification.

[21] Bouril A., Aleinikava D., Guillem M. S., Mirsky G. M. Automated classification of normal and abnormal heart sounds using support vector machines.

[22] Gonzalez Ortiz J. J., Phoo C. P., Wiens J. Heart Sound Classification Based on Temporal Alignment Techniques.

[23] Nabhan Homsi M., Medina N., Hernandez M. Automatic Heart Sound Recording Classification using a Nested Set of Ensemble Algorithms.

[24] Langley P., Murray A. (2017). Heart sound classification from unsegmented phonocardiograms. *Physiological Measurement*.

[25] Nabhan Homsi M., Warrick P. (2017). Ensemble methods with outliers for phonocardiogram classification. *Physiological Measurement*.

[26] Whitaker B., Anderson D. Heart Sound Classification via Sparse Coding.

[27] Whitaker B. M., Suresha P. B., Liu C., Clifford G. D., Anderson D. V. (2017). Combining sparse coding and time-domain features for heart sound classification. *Physiological Measurement*.

[28] Potes C., Parvaneh S., Rahman A., Conroy B. Ensemble of feature-based and deep learning-based classifiers for detection of abnormal heart sounds.

[29] Zabihi M., Bahrami Rad A., Kiranyaz S., Gabbouj M., K. Katsaggelos A. Heart Sound Anomaly and Quality Detection using Ensemble of Neural Networks without Segmentation.

[30] Kay E., Agarwal A. DropConnected Neural Network Trained with Diverse Features for Classifying Heart Sounds.

[31] Tang H., Chen H., Li T., Zhong M. Classification of Normal/Abnormal Heart Sound Recordings based on Multi:Domain Features and Back Propagation Neural Network.

[32] http://www.physionet.org/challenge/2016/, Nov. 16, 2016

[33] Liu C., Springer D., Li Q. (2016). An open access database for the evaluation of heart sound algorithms. *Physiological Measurement*.

[34] Schmidt S. E., Holst-Hansen C., Graff C., Toft E., Struijk J. J. (2010). Segmentation of heart sound recordings by a duration-dependent hidden Markov model. *Physiological Measurement*.

[35] Springer D. B., Tarassenko L., Clifford G. D. (2016). Logistic regression-HSMM-based heart sound segmentation. *IEEE Transactions on Biomedical Engineering*.

[36] Boucheron L. E., De Leon P. L., Sandoval S. (2012). Low bit-rate speech coding through quantization of mel-frequency cepstral coefficients. *IEEE Transactions on Audio, Speech and Language Processing*.

[37] Li T., Tang H., Qiu T., Park Y. (2011). Best subsequence selection of heart sound recording based on degree of sound periodicity. *IEEE Electronics Letters*.

[38] Liu C., Li K., Zhao L. (2013). Analysis of heart rate variability using fuzzy measure entropy. *Computers in Biology and Medicine*.

[39] Zhao L., Wei S., Zhang C. (2015). Determination of sample entropy and fuzzy measure entropy parameters for distinguishing congestive heart failure from normal sinus rhythm subjects. *Entropy*.

